# Dural Changes Induced by an Ultrasonic Bone Curette in an Excised Porcine Spinal Cord

**DOI:** 10.3390/vetsci9110601

**Published:** 2022-10-28

**Authors:** Rizou Ota, Eri Iwaki, Kentaro Sakai, Tomohiro Haraguchi, Yasuyuki Kaneko, Satoshi Sekiguchi, Ryoji Yamaguchi, Kiyokazu Naganobu

**Affiliations:** 1 Veterinary Teaching Hospital, Faculty of Agriculture, University of Miyazaki, Miyazaki 889-2192, Japan; 2 Inuyama Animal General Medical Center, 29, Haguro Omishita, Inuyama-shi 484-0894, Japan; 3 Division of Materials Research, Center for Collaborative Research & Community Cooperation, University of Miyazaki, Miyazaki 889-2192, Japan; 4 Technical Center, Faculty of Engineering, University of Miyazaki, Miyazaki 889-2192, Japan; 5 Animal Infectious Disease and Prevention, Department of Veterinary Sciences, Faculty of Agriculture, University of Miyazaki, Miyazaki 889-2192, Japan; 6 Veterinary Pathology, Department of Veterinary Sciences, Faculty of Agriculture, University of Miyazaki, Miyazaki 889-2192, Japan

**Keywords:** cotton pattie, dura, spinal cord, ultrasonic bone curette

## Abstract

**Simple Summary:**

An ultrasonic bone curette is a surgical device used for bone removal during spinal surgery. This curette is considered unlikely to cause mechanical injury to the dura, a thick membrane surrounding the spinal cord, when it accidentally makes contact with the dura. We investigated the effect of direct contact with an ultrasonic bone curette on the dura and the protective effect of covering the dura with a piece of cotton using an excised porcine spinal cord. The ultrasonic bone curette was pressed against the porcine spinal cord and activated for 1 s, with or without covering the dura with a piece of cotton. None of the specimens showed dural perforation, although non-perforating injuries to the dura were observed in many specimens. Histological changes were also observed in some of the specimens. The use of cotton reduced the occurrence of these changes, but it did not prevent them when ultrasonic vibration was applied with a large force. We considered ultrasonic bone curettes to have a low risk of dural perforation and, thus, to be a safe surgical device as long as they did not accidentally make strong contact with the dura.

**Abstract:**

In spinal surgery, ultrasonic bone curettes are considered unlikely to cause mechanical injury to the dura; however, there is little evidence to support this claim. We investigated the effect of direct contact with an ultrasonic bone curette on the dura and the protective effect of covering the dura with a cotton pattie using an excised porcine spinal cord. The ultrasonic bone curette was pressed against the porcine spinal cord with constant force and activated for 1 s, with or without covering the dura with a cotton pattie. The dural surface and cross-section were observed using electron and light microscopy. When the ultrasonic bone curette was applied directly against the dura, most specimens showed non-perforating dural injuries. However, none of the specimens showed dural perforation. Histological changes were also observed. The use of a cotton pattie reduced the occurrence of these changes, although it did not prevent them when ultrasonic vibration was applied with a large force. We considered ultrasonic bone curettes to have a low risk of dural perforation and, thus, to be a safe surgical device as long as they did not accidentally make strong contact with the dura.

## 1. Introduction

An ultrasonic bone curette is a surgical device that can cut and scrape bone by vibrating at an ultrasonic frequency and is widely used in spinal, cranial, oral, orbital, and maxillofacial surgeries in humans [1,2,3,4,5,6,7,8,9]. In veterinary spinal surgery, high-speed drills are widely used to remove bone [10,11,12]. In recent years, ultrasonic surgical devices have also been used for bone cutting in various neurosurgeries in dogs and cats [13].

In a meta-analysis in human spinal surgery [14], no significant difference was found between ultrasonic bone curettes and conventional surgical equipment in symptom improvement rate, length of hospital stay, or postoperative complications. However, the possible advantages of ultrasonic bone curettes over high-speed drills in humans include decreased risk of mechanical injury to soft tissues such as the dura mater, nerve roots, or spinal cord [15,16,17,18]; less bleeding during surgery [1,19,20]; and shorter surgery time [20]. In addition, it is possible to reduce the soft tissue damage caused by ultrasonic bone curettes by covering the adjacent soft tissues with a cotton pattie [4,15,18,21,22]. Because high-speed drills entangle cotton patties [15], the dura mater cannot be protected by a cotton pattie when using a high-speed drill.

The use of ultrasonic bone curettes during spinal surgery in humans also causes dural injuries such as dural tears or thermal injuries [16,21,23,24,25]. In addition, ultrasonic bone curettes have been reported to reach higher temperatures than high-speed drills during bone cutting [26,27,28]. The heat generated by a high-speed drill during bone cutting may injure the nerve roots and other tissues adjacent to the bone [29,30]. Therefore, although accidental contact of the dura with the ultrasonic bone curette may cause only mild mechanical injury, there is a potential for damage to the dura or spinal cord due to temperature. 

However, there is little information on injuries to the dura mater caused by direct contact with an ultrasonic bone curette or the protective effect of a cotton pattie. This study aimed to evaluate the effect of a short period of direct contact with an ultrasonic bone curette on the dura mater and the protective effect of covering the dura with a cotton pattie using an excised porcine spinal cord.

## 2. Materials and Methods

### 2.1. Experiment 1

This study was approved by the Animal Experiment Committee of the University of Miyazaki, Japan (No. 2018-008). The porcine spinal cords used in the experiments were obtained from a slaughterhouse. The spinal cords were removed from the pigs slaughtered on the morning of the experiment, stored under refrigeration, and tested on the same day.

**Figure 1:**
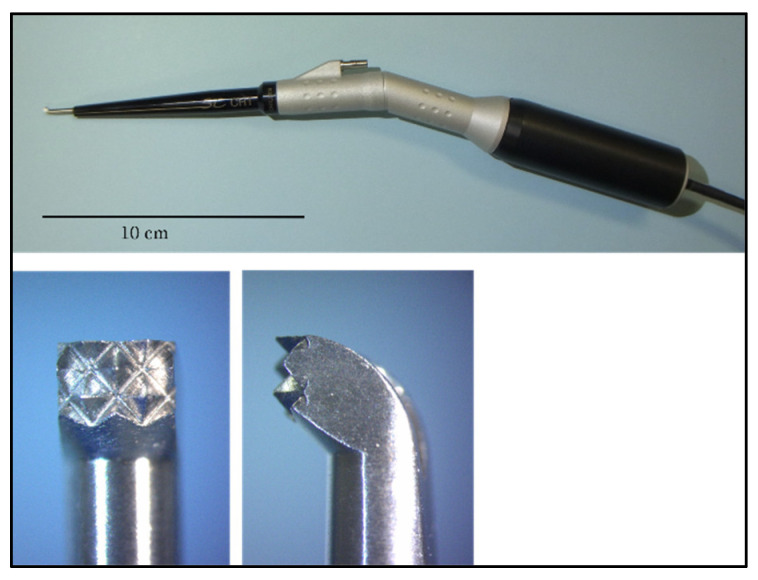
Ultrasonic bone curette handpiece (top photo) and the bone tip (bottom two photos). The width of the tip is 2 mm.

**Figure 2:**
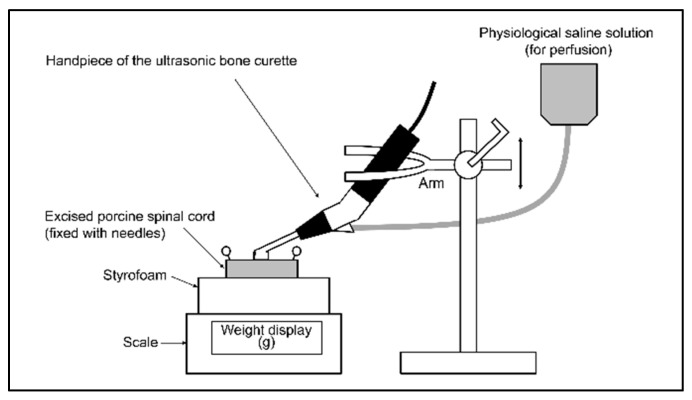
Schematic diagram of the experimental apparatus. A block of hard Styrofoam was placed on the scale, and the spinal cord specimen was fixed with needles. The ultrasonic bone curette handpiece was securely fixed to the arm of the stand, and the height of the arm was adjusted such that the ultrasonic bone curette pressed on the spinal cord specimen from above to yield a scale reading of 2, 10, or 50 g. The entire cutting surface of the handpiece tip was in contact with the spinal cord.

The ultrasonic bone curette used in this study was SonoCure^®^ (Tokyo Iken Co., Ltd., Inagi, Tokyo, Japan). A bone tip, CH1^®^ (width of the tip: 2 mm; Figure 1), was attached to the handpiece of the curette. This tip vibrates in the longitudinal and torsional directions at 25 kHz and is designed to operate while irrigating and aspirating with saline to attenuate temperature rise. The device settings used were as follows: ultrasonic power, 70%; irrigation rate, 10 mL/min; suction flow rate, 7.0 ± 0.5 L/min; and maximum suction pressure, 30 kPa. These settings are frequently used in spinal surgery in dogs. The ultrasonic feature of the curette is operated by a foot switch; however, for this experiment, we modified the machine such that stepping on the switch would enable it to operate for a period of 1 s only. Figure 2 shows the schematic diagram of the experimental apparatus. A block of hard Styrofoam was placed on a scale (Miniscale^®^ MS-2000; Custom, Tokyo, Japan), and a spinal cord specimen cut to a length of 2 cm was fixed to the Styrofoam with needles. The spinal cord specimen was marked with a tissue dye (Tissue Marking Dye^®^; Falma, Tokyo, Japan) on the dura mater to indicate the area where the tip of the curette would make contact. The handpiece of the ultrasonic bone curette was securely fixed to the arm of the stand. The arm height was then adjusted and fixed such that the hand piece of the curette was pressed against the spinal cord specimen from above, causing the scale to read approximately 2 g (range: 1–3 g), 10 g (range: 9–11 g), or 50 g (range: 49–51 g). These levels are hereafter abbreviated as 2 g load, 10 g load, and 50 g load, respectively. The tip of the handpiece was positioned such that the entire cutting surface was in contact with the dura mater. The samples were divided into two groups: the uncovered group, in which the bone tip was in direct contact with the dura, and the covered group, in which the dorsal surface of the dura was covered with a neurosurgical cotton pattie (NonstinaX^®^; Hakujuji Co., Ltd., Tokyo, Japan). Experiments were conducted under the following six conditions (*n* = 4 each): 2 g load uncovered, 10 g load uncovered, 50 g load uncovered, 2 g load covered, 10 g load covered, and 50 g load covered. Neurosurgical cotton patties were pre-moistened with a saline solution. Finally, under all conditions, the ultrasonic bone curette was activated for 1 s using a modified foot switch.

### 2.2. Evaluation Using a Scanning Electron Microscope and Stereomicroscope

Spinal cord specimens were fixed in 2.5% glutaraldehyde solution for 27–41 h and immersed in ethanol, followed by dehydration with tert-butyl alcohol. The samples were then placed in a dry chamber, frozen, and vacuum freeze-dried using an ES-2030 vacuum freeze-dryer (Hitachi High Technologies, Tokyo, Japan). An ion-sputtering system (E-1030; Hitachi, Tokyo, Japan) was used to coat the samples with an Au film of 8.0 nm thickness to achieve conductivity. The dural surface was observed using a scanning electron microscope (SEM; SU3500; Hitachi, Tokyo, Japan). The acceleration voltage was set to 5.0 kV.

Following SEM observation, the spinal cord was cut using a scalpel under a stereomicroscope to visualize a transverse section of the site where SEM showed the biggest changes in dura mater tissue. The cut surface was observed using a stereomicroscope (SZX10; Olympus, Tokyo, Japan). The results of electron microscopy and stereomicroscopy were combined to evaluate the presence or absence of dural rupture or perforation, dural indentation in the absence of rupture or perforation (i.e., non-perforating dural wounds), and discoloration of the dural cross-section.

### 2.3. Histological Examination

Stereomicroscopic observation of the dural cross-section revealed specimens with partial dark brown discoloration (hereafter referred to as “discoloration”) of the dura. Two spinal cord specimens from each experimental condition were selected for histological examination of the discolored sites. In particular, histological examination was performed for six specimens with dura mater discoloration on stereomicroscopic observation (10 g load uncovered [*n* = 2], 50 g load uncovered [*n* = 2], 50 g load covered [*n* = 2]) and six specimens without discoloration on stereomicroscopic observation (2 g load uncovered [*n* = 2], 2 g load covered [*n* = 2], and 10 g load covered [*n* = 2]). Spinal cord specimens were fixed in 10% neutral-buffered formalin and embedded in paraffin. Sections of 3–5-μm thickness were prepared and stained with hematoxylin and eosin. A veterinary pathologist (R.Y.) performed the histological examinations.

### 2.4. Experiment 2

Ultrasonic bone curette and spinal cord specimens were prepared in the same manner as in Experiment 1. However, in Experiment 2, the bone tip was simply pressed onto the spinal cord specimen without using ultrasound features. Experiment 2 used the same six conditions as Experiment 1 (*n* = 3 each): 2 g load uncovered, 10 g load uncovered, 50 g load uncovered, 2 g load covered, 10 g load covered, and 50 g load covered. Subsequently, electron microscopy, stereomicroscopy, and histological examinations were performed as in Experiment 1. For the histological examination, two specimens were randomly selected from each experimental condition.

### 2.5. Untreated Specimens

Electron microscopy, stereomicroscopy, and histological examination were also performed on the control group of untreated spinal cord specimens (*n* = 4).

### 2.6. Statistical Analysis

Fisher’s exact test was performed to compare the incidence of dural indentation and discoloration between the two groups. To compare the incidence of dural indentation and discoloration across the three groups, Fisher’s exact test was Bonferroni-corrected for multiple post hoc comparisons. EZR (ver. 1.54, 24 December 2020) was used for all statistical analyses. The significance level was set at *p* < 0.05.

## 3. Results

### 3.1. Changes in the Spinal Cord When Pressed by the Ultrasonic Bone Curette

**Figure 3:**
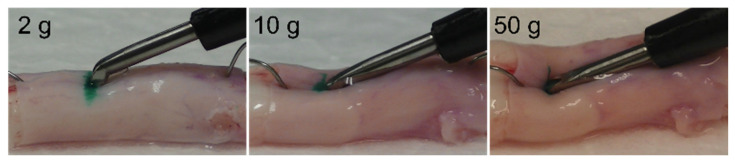
Examples of changes in the spinal cord when pressed by the ultrasonic bone curette at a scale reading of 2, 10, and 50 g.

Figure 3 shows examples of the changes in the dura mater when the ultrasonic bone curette was pressed onto the spinal cord specimens without ultrasonic vibration. The spinal cord specimen was slightly indented when 2 g of pressure was applied with a curette, and the specimens were clearly indented at 10 g and 50 g of applied pressure.

### 3.2. Dural Changes Induced by Ultrasonic Vibration

**Figure 4:**
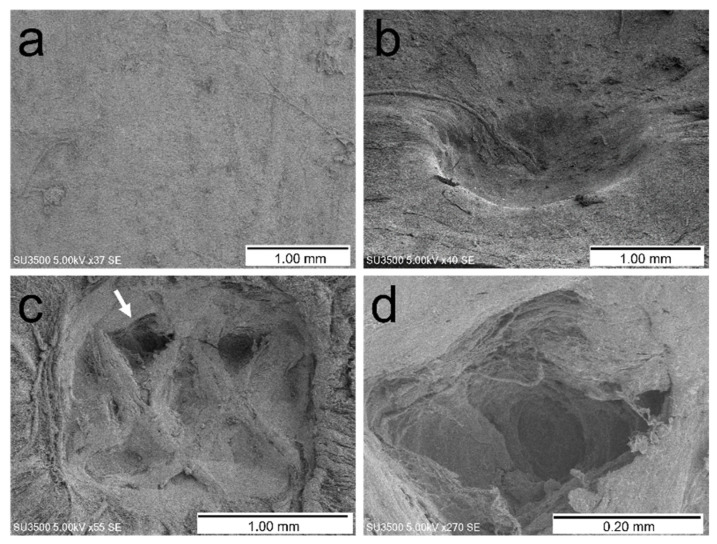
Examples of changes observed in the dura mater with electron microscopy. (**a**) Control. (**b**) Dural indentation (50 g load, covered with neurosurgical cotton pattie). (**c**) Dural indentation or partial non-perforating wound resembling the shape of the ultrasound tip (50 g load, uncovered). (**d**) Partial enlargement of the site indicated by the arrow in image (**c**).

**Figure 5:**
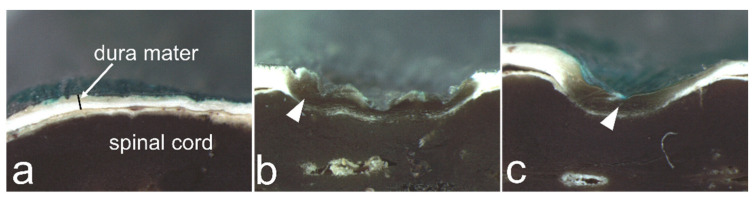
Examples of changes observed in the dura mater with stereomicroscopy. (**a**) Control. (**b**) Dural indentation and dark brown discoloration (arrowhead; 50 g load, no covering). (**c**) Dural indentation and dark brown discoloration (arrowhead; 50 g load, covered with a neurosurgical cotton pattie).

Table 1 summarizes the effects of ultrasonic vibrations on the dura mater. No ruptures or perforations were observed. Electron microscopy and stereomicroscopy of the sonicated specimens revealed dural indentation and non-perforating wounds (Figure 4), as well as partial dark brown discoloration of the dura mater in the cross-section (Figure 5). The shapes of the dural indentations and non-perforating wounds varied, including a bowl-shaped indentation (Figure 4b) and an indentation resembling the shape of the ultrasound tip (Figure 4c,d). Since it is sometimes difficult to distinguish between dural indentations and non-perforating wounds, we refer to them collectively as dural indentations hereafter.

In the uncovered group, dural indentation was present in all sonicated specimens, except for a 2 g load specimen (Table 1). Furthermore, when the dura mater was not covered, dural discoloration was observed in one of the four specimens under the 2 g load condition and in all specimens under the 10 g and 50 g load conditions (Table 1). In contrast, when the dura was covered with a neurosurgical cotton pattie, dural indentation or discoloration was not observed in any specimen at 2 g or 10 g load, but it was observed in all specimens at 50 g load (Table 1). When the dura mater was covered with a cotton pattie, the number of specimens with dural indentation and discoloration was significantly higher at 50 g load than at 2 g or 10 g load. At a load of 10 g, covering the dura mater with a neurosurgical cotton pattie significantly reduced the incidence of both indentation and discoloration. In the uncovered condition, the percentage of specimens with discoloration did not differ significantly between the 2 g, 10 g, and 50 g load conditions, but there was a trend toward a lower rate of discoloration at the 2 g load (*p* = 0.0545).

### 3.3. Dural Changes When the Spinal Cord Is Pressed without Ultrasonic Vibration

Table 2 shows the changes in the dura mater at 2 g, 10 g, and 50 g load conditions without the use of ultrasonic vibration. When the dura mater was not covered, dural indentation was not observed for the 2 g load, but it was observed for the 10 g and 50 g loads. The number of specimens showing dural indentation was significantly higher at 10 g and 50 g loads than at 2 g load when the dura mater was not covered. No dural discoloration was observed in any specimen. When the dura mater was covered with a neurosurgical cotton pattie, indentation or discoloration was not observed in any specimen.

When the dura mater was uncovered, the sonicated group had a significantly higher incidence of dural discoloration than the non-sonicated group under 10 g and 50 g load conditions (*p* = 0.0286 for both). When the dura mater was covered by a neurosurgical cotton pattie, the sonicated group had a significantly higher incidence of dural indentation and discoloration than the non-sonicated group at a 50 g load (*p* = 0.0286 for both). 

### 3.4. Histological Findings

**Figure 6:**
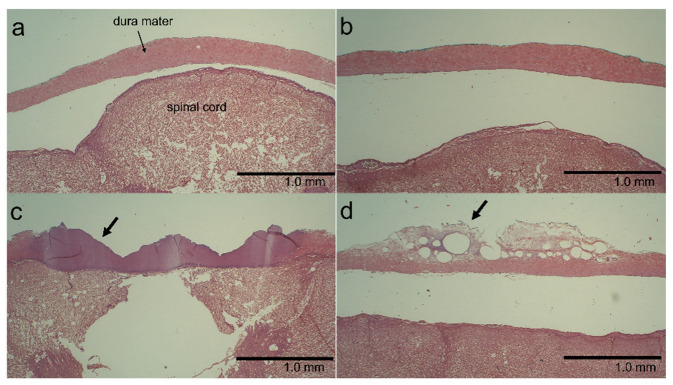
Examples of histological changes in the dura mater. (**a**) Control. (**b**) No noteworthy change is seen (10 g load, covered with a neurosurgical cotton pattie). (**c**) Coagulative necrosis-like change (arrow; 50 g load, uncovered). (**d**) Vesicle formation with edema (arrow; 2 g load, uncovered).

Six specimens that exhibited dural discoloration after ultrasonic vibration were selected from the specimens listed in Table 1. In five of these specimens, histological changes were noted that were relatively astructural and eosinophilic (10 g load uncovered [*n* = 1], 50 g load covered [*n* = 2], and 50 g load uncovered [*n* = 2]; Figure 6c). Since this histological change resembled the coagulative necrosis that occurs in vivo, we have hereafter referred to this histological change as “coagulative necrosis-like change” for convenience. The remaining specimens showed vesicle formation with edema (10 g load uncovered). Of the six specimens selected from those without discoloration, two showed vesicle formation with edema (2 g load uncovered [*n* = 2]; Figure 6d), whereas the other four showed no noteworthy change (2 g load covered [*n* = 2] and 10 g load covered [*n* = 2]; Figure 6b). No noteworthy change was observed in the dura mater of specimens that did not undergo ultrasonic vibration (Table 2).

## 4. Discussion

The primary findings of this study are as follows: (1) the ultrasonic bone curette did not cause rupture or perforation of the dura mater; (2) ultrasonic bone curettes may cause dural injuries such as indentation, non-perforating wounds, or dural tissue changes; (3) injury to the dura mater caused by ultrasonic bone curettes can be reduced using a neurosurgical cotton pattie; and (4) injury to the dura mater can occur even with a neurosurgical cotton pattie if the ultrasonic bone curette is pressed with sufficient force.

Previous in vitro studies using other types of ultrasonic surgical instruments on bovine and porcine bones reported loads of 151–328 gram-force (gf) applied to the bone during cutting [31,32]. Therefore, if the ultrasonic surgical instrument accidentally contacts the dura mater, it may be pressed onto the dura mater with a load of 151–328 gf. In contrast, in clinical situations, when the ultrasonic bone curette accidentally comes in contact with the spinal cord, it is usually pulled away; therefore, under normal circumstances, it is unlikely that the curette would continue to push the dura mater and spinal cord with the same force as when cutting the bone.

In the present study, when the spinal cord specimen was pressed in such a way that the scale read 2 g, the specimen was indented slightly; however, at 50 g, the specimen became distinctly indented (Figure 3). Therefore, we established experimental conditions up to a reading of 50 g. In addition, we modified the device to operate an ultrasonic bone curette for a short time (1s). Therefore, the contact conditions used in our experiment appear to be within the range of what could actually occur. However, the spinal cord can be pressed with a force greater than that used in the present study. Furthermore, when an ultrasonic bone curette is used to cut or scrape the spine, it can contact the spine at various angles, and the area where the tip contacts the dura mater can vary. In this study, the entire surface of the blade was in contact with the dura mater because the contact point was most likely to cause damage to the dura mater.

In the present study, no rupture or perforation of the dura mater occurred, even when the ultrasonic bone curette was pressed against the spinal cord specimen with sufficient force to cause considerable indentation. This observation supports the notion that an ultrasonic bone curette is less likely to cause mechanical injury to soft tissues such as the dura mater [15,16,17,18]. This is also consistent with the finding that piezosurgery, another type of ultrasonic surgery, cuts the bone but not the soft tissue of the tongue and only indents its surface in rats [33]. 

In contrast, although the present experiments did not result in rupture of the porcine dura mater, they caused dural indentation, non-perforating wounds, and histologically, coagulative necrosis-like change and vesicle formation with edema. It seems likely that if the dura mater is thin or fragile, or if the ultrasonic bone curette has abnormally forceful or prolonged contact with the dura mater, rupture and perforation of the dura mater may occur. 

In the present study, coagulative necrosis-like change and vesicle formation with edema were observed in the dura mater of most specimens with dural discoloration. Thus, dural discoloration seen under stereomicroscopy would suggest the development of histological changes. Suetsuna et al. showed in dogs that placing an ultrasonic surgical aspirator, a device that uses ultrasonic vibration to remove spinal or spinal cord tumors, in light contact with the dura mater and operating it for 10–20 s induced coagulative necrosis, swelling, and loosening of the collagen tissue in the dura mater [34]. The histological changes observed in the present study are similar to those reported by Suetsuna et al.

Suzuki et al. investigated changes in bone temperature during bone curetting of a porcine skull using an ultrasonic bone curette. They reported that temperature changes differed depending on factors such as cutting method and irrigation rate. For example, bone temperature reached a maximum of 85.6 °C when bone cutting was performed with an irrigation rate of 10 mL/min, and it reached a maximum of 203.0 °C when the irrigation rate was reduced [27]. Other studies have reported that ultrasonic bone curettes reach higher temperatures than high-speed drills [26,28]. Heat can cause coagulative necrosis in the tissues. Therefore, although we did not obtain temperature measurements, coagulative necrosis-like change in the dura mater in the present study may have been caused by the heat generated by the ultrasonic bone curette. On the other hand, it is unclear whether the same degree of heat is generated when ultrasonic bone curette is used on dura as it is on bone. The temperature change when the ultrasonic bone curette makes direct contact with the dura mater should also be studied in the future.

Previously, attempts have been made to increase the irrigation flow rate or to use cold irrigation solutions to reduce the heat generated by ultrasonic bone curettes or high-speed drills [26,27,28,35]. These methods may be considered to reduce dural damage in cases where the ultrasonic bone curette accidentally contacts the dura. 

In this study, we used an irrigation flow rate of 10 mL/min. Crovace et al. used an irrigation flow rate of 30 mL/min for an ultrasonic surgical device for bone cutting in various neurosurgeries in dogs and cats [13]. Thus, irrigation flow rates greater than 10 mL/min, such as 30 mL/min, may be used to decrease heat generation.

In the present study, when the dura mater was not covered, dural indentation and discoloration were observed in all specimens, for which the dura mater was pressed at a force of 2–50 g and ultrasonic vibration was applied, excluding some cases pressed at 2 g load conditions. When the spinal cord specimens were subjected to ultrasonic vibration while the dura mater was covered with a neurosurgical cotton pattie, no dural indentation or discoloration occurred under the 2 g and 10 g load conditions. Therefore, a neurosurgical cotton pattie can provide a protective effect, particularly in cases of light contact between the ultrasonic bone curette and the dura mater. However, at a load of 50 g, the incidence of dural indentation and discoloration was the same with and without the neurosurgical cotton pattie. Therefore, when the ultrasonic bone curette is forcefully pressed against the dura, a neurosurgical cotton pattie cannot protect the dura mater from damage such as indentation or histological injury. To the best of our knowledge, the protective effect of cotton patties on the dura has not been well studied in dogs and cats. Further research is needed to determine the protective effect of the cotton pattie in vivo.

The present study is limited in that the experiments were conducted using spinal cords removed from slaughtered pigs rather than living animals. However, as the changes in the dura mater observed in this study were likely due to compression, vibration, or heat, similar dural changes are expected to occur in living animals. The histological changes (coagulative necrosis-like change and vesicle formation with edema) observed in the dura mater were similar to those observed in a previous in vivo study in dogs [34]. However, whether the present results also apply to in vivo conditions remains to be determined. Additionally, it is possible for an ultrasonic bone curette to contact the dura mater from various angles. Our experiments used the contact angle most likely to injure the dura mater. However, contact from different angles or surfaces of the curette could cause different dural changes than those observed in the present study. We did not examine the effects of ultrasonic bone curette on the neighboring spinal cord in the present study. Effects on the neighboring spinal cord should also be evaluated in the future.

## 5. Conclusions

In excised porcine spinal cords, we found (1) the ultrasonic bone curette did not cause rupture or perforation of the dura mater; (2) ultrasonic bone curettes may cause dural injuries such as indentation, non-perforating wounds, or dural tissue changes; (3) injury to the dura mater caused by ultrasonic bone curettes can be reduced using a neurosurgical cotton pattie; and (4) injury to the dura mater can occur even with a neurosurgical cotton pattie if the ultrasonic bone curette is pressed with sufficient force. Thus, the risk of rupture or perforation of the dura mater is low even when the ultrasonic bone curette makes direct contact with the dura mater. However, depending on the conditions under which the ultrasonic bone curette comes into contact with the dura mater, physical damage or heat-induced tissue changes may occur in the dura mater. Increasing the irrigation flow rate or using a cold irrigation solution may reduce heat generation when the ultrasonic bone curette accidentally contacts the dura. The use of cotton patties can reduce the incidence of injury to the dura mater. However, the dura mater can be injured if the ultrasonic bone curette is pressed forcefully. Therefore, we considered the ultrasonic bone curette to be a safe surgical device as long as it did not accidentally make strong contact with the dura. Future in vivo studies are needed to confirm these results. Our findings can serve as observational data and provide experimental conditions for future research using living animals. Since we did not obtain temperature measurements, the temperature change when the ultrasonic bone curette makes direct contact with the dura mater should also be studied in the future.

## Figures and Tables

**Table 1:** Changes in the dura mater from ultrasonic bone curette oscillation (electron microscopy and stereomicroscopy).

Scale Display (g)	Spinal Dural Covering	*n*	No. of Specimens with Dural Changes		
			Penetration	Dural Indentation (Depressions or Blade Marks)	Discoloration
2	No covering	4	0 (0)	3 (75)	1 (25)
	Cotton covering	4	0 (0)	0 (0)	0 (0)
10	No covering	4	0 (0)	4 (100)	4 (100)
	Cotton covering	4	0 (0)	0 (0) *	0 (0) *
50	No covering	4	0 (0)	4 (100)	4 (100)
	Cotton covering	4	0 (0)	4 (100) ^§^	4 (100) ^§^

Penetration values are expressed as number (%). * Significant difference (*p* < 0.05) from the spinal covering group on the same scale (g). ^§^ Significant difference from 2 g and 10 g load conditions when using a cotton pattie covering the spinal dura mater (*p* < 0.05).

**Table 2:** Changes in the dura mater from the ultrasonic bone curette pressing the spinal cord without ultrasonic oscillation (electron microscopy and stereomicroscopy).

Scale Display (g)	Spinal Dural Covering	*n*	No. of Specimens with Dural Changes		
			Penetration	Dural Indentation (Depressions or Blade Marks)	Discoloration
2	No covering	3	0 (0)	0 (0)	0 (0)
	Cotton covering	3	0 (0)	0 (0)	0 (0)
10	No covering	3	0 (0)	3 (100) *	0 (0)
	Cotton covering	3	0 (0)	0 (0)	0 (0)
50	No covering	3	0 (0)	3 (100) *	0 (0)
	Cotton covering	3	0 (0)	0 (0)	0 (0)

Penetration values are expressed as number (%). * Significant difference from the 2 g load condition when the dura mater was not covered (*p* < 0.05).

## Data Availability

Not applicable.
